# The dose-dependent efficiency of radial shock wave therapy for patients with carpal tunnel syndrome: a prospective, randomized, single-blind, placebo-controlled trial

**DOI:** 10.1038/srep38344

**Published:** 2016-12-02

**Authors:** Ming-Jen Ke, Liang-Cheng Chen, Yu-Ching Chou, Tsung-Ying Li, Heng-Yi Chu, Chia-Kuang Tsai, Yung-Tsan Wu

**Affiliations:** 1 Department of Physical Medicine and Rehabilitation, Tri-Service General Hospital, School of Medicine, National Defense Medical Center, No. 325, Sec. 2, Cheng-Kung Road, Neihu District, Taipei, Taiwan, Republic of China; 2 School of Public Health, National Defense Medical Center, No. 161, Sec. 6, Minquan East Road, Neihu District, Taipei, Taiwan, Republic of China; 3 Department of Neurology, Tri-Service General Hospital, School of Medicine, National Defense Medical Center, No. 325, Sec. 2, Cheng-Kung Road, Neihu District, Taipei 11490, Taiwan, Republic of China; 4 Graduate Institute of Medical Science, School of Medicine, National Defense Medical Center, No. 161, Sec. 6, Minquan East Road, Neihu District, Taipei, Taiwan, Republic of China

## Abstract

Recently, extracorporeal shock wave therapy (ESWT) has been shown to be a novel therapy for carpal tunnel syndrome (CTS). However, previous studies did not examine the diverse effects of different-session ESWT for different-grades CTS. Thus, we conducted a randomized, single-blind, placebo-controlled study. Sixty-nine patients (90 wrists) with mild to moderate CTS were randomized into 3 groups. Group A and C patients received one session of radial ESWT (rESWT) and sham eESWT per week for 3 consecutive weeks, respectively; Group B patients received a single session of rESWT. The night splint was also used in all patients. The primary outcome was Boston Carpal Tunnel Syndrome Questionnaire (BCTQ) points, whereas secondary outcomes included the sensory nerve conduction velocity and cross-sectional area of the median nerve. Evaluations were performed at 4, 10, and 14 weeks after the first session of rESWT. Compared to the control group, the three-session rESWT group demonstrated significant BCTQ point reductions at least 14 weeks, and the effect was much longer lasting in patients with moderate CTS than mild CTS. In contrast, the effect of single-session rESWT showed insignificant comparison. rESWT is a valuable strategy for treating CTS and multiple-session rESWT has a clinically cumulative effect.

Carpal tunnel syndrome (CTS), which results from compression of the median nerve as it passes through the carpal tunnel, is the most common focal entrapment neuropathy. The estimated prevalence of CTS is about 1 to 5% in the general population, and is more frequent in women (0.7 to 9.2%) than in men (0.4 to 2.1%)^1^,^2^. The characteristic symptoms and signs of CTS are numbness, tingling pain, paresthesia of at least 2 of 3 digits, and a burning sensation in the area innervated by the median nerve, with nocturnal paresthesia, and sometimes thenar muscle atrophy as seen in severe cases^1^. There are a number of risk factors associated with CTS including overuse of the wrist with repetitive stress, obesity, female gender, pregnancy, diabetes, rheumatoid arthritis, hypothyroidism, and connective tissue diseases^1^,^3^. The pathophysiology of CTS is thought to be multifactorial, mainly involving ischemic changes and mechanical injury resulting from increased pressure in the intra-carpal canal. Indeed, Gelberman *et al*. found a significantly higher pressure in the intra-carpal canal in patients with CTS compared with patients without CTS (32 mmHg vs. 2.5 mmHg)^4^.

There are many interventions for treating CTS, including a conservative strategy (wrist splint, steroid injections or therapeutic ultrasound etc.), and surgical decompression of the median nerve. Although surgical intervention is more effective than conservative treatment, conservative therapies are advocated for mild to moderate CTS, and surgical therapy is suggested for severe CTS or patients with a poor response from conservative treatments, because the failure rate of surgery ranges from 7 to 75% with recurrence of symptoms, persistent neurophysiologic findings, or associated complications^5^,^6^,^7^,^8^. On the other hand, around 60–70% of conservatively treated patients with CTS remained symptomatic after 18 months^9^. Hence, it is important to find a novel intervention for CTS before surgery is undertaken.

Extracorporeal shock wave therapy (ESWT) are a transient sequence of acoustic pulses with a high peak pressure (100 Mpa), followed by a negative pressure about of 5–10 Mpa, with an energy density between 0.003–0.89 mJ/mm^2^ 10. ESWT is classified into focused ESWT (fESWT) and defocused (radial) ESWT (rESWT) based on the design of the reflector for its pressure field and energy. The characteristics of rESWT are less penetrative depth, less focusing of the energy to a targeted spot, and relatively lower intensity compared with fESWT^11^. ESWT has been extensively clinically applied in treating miscellaneous musculoskeletal disorders like plantar fasciitis, chronic calcifying tendonitis, and lateral epicondylitis^10^,^12^.

Recently, both fESWT and rESWT have received increased attention as being a safe and novel therapy for CTS^11^,^13^,^14^,^15^. In 2015, we reported the first prospective, randomized, double-blind, placebo-controlled study, and revealed the effective benefit of rESWT for treating CTS after 3 months follow-up^11^. However, the evidence for ESWT in CTS from current published studies is not as yet available because of small patient numbers and the lack of a placebo-controlled design in most studies. In addition, whether varying the number of ESWT sessions would affect the duration of the therapeutic effect has not been investigated in a single study. Moreover, previous studies did not identify in detail the grading of CTS, and further survey the diverse effects of ESWT for different grades of CTS. Thus, we performed a larger, prospective, randomized, single-blind, placebo-controlled study to investigate the dose-dependent effect of rESWT in patients with mild to moderate CTS.

## Methods

### Study design

This study was designed according to the CONSORT 2010 statement^16^. This was a prospective, randomized, placebo-controlled, single-blind study conducted in a single medical center from August 2014 to March 2016. It was reviewed and approved by the institutional review board of the Tri-Service General Hospital (No. 1-102-05-122) and all enrolled subjects have given their fully-informed written consent for this study with the registration number NCT02218229 at ClinicalTrials. gov on 08/13/2014. All methods for each subject were performed in accordance with the approved ethical guideline and there was no changes made to this trial after the commencement of the recruitment. Ninety patients diagnosed with CTS were screened for eligibility; 69 were enrolled into this study, and all were block randomized with a 1:1:1 ratio into 3 groups by an independent researcher with the use of study numbers generated by computer randomization (Microsoft Excel, Microsoft Inc., USA). If the patients had bilateral CTS, the same dose of ESWT was prescribed in each wrist.

In group A, participants received one session of rESWT per week for 3 consecutive weeks; in Group B, the participants received one single session of rESWT; Group C participants received one session of sham rESWT at the same intervals as group A. In order to maintain fundamental care, a wrist night splint was provided for all participants in the three groups since first-session of rESWT. The wrist night splint was firmly fixed in a neutral position to immobilize the affected wrist, and participants were informed to wear the splint at night for at least 8 hours during the whole period of this study^11^,^17^. All participants were instructed to refrain from alternative treatments for the discomfort of CTS including analgesic drugs, acupuncture therapy, manual therapy, ultrasound/laser therapy, or any nonsurgical therapy for CTS, from 1 week prior to participation to the end of the follow-up period. The patients were also required to inform us if any of those therapies had been used.

### Inclusion and exclusion criteria

All participants could be considered and enrolled if they conformed to the diagnosis of CTS, with clinical symptoms for at least 3 months, and proof from electrophysiological studies. The clinical symptoms and signs for the diagnosis of CTS were as follows: (1) paresthesias and painful swelling with clumsy weakness of the affected hand, exacerbated while sleeping or by repetitive use of the wrist, which would be relieved by shaking the hand with postural change; (2) sensory loss with numbness in the regions of the hand innervated by the median nerve; (3) impaired motor function with atrophy of the median nerve-innervated thenar muscles; and (4) positive Phalen’s test and/or Tinel’s sign. If the subject met criterion 1, and one or more of criteria 2–4, the clinical diagnosis of CTS was confirmed^18^,^19^.

The electrophysiological study of CTS with cut-off points for normal values in our study were as follows: (1) the upper limit of median sensory nerve distal latency is ≤3.6 ms at a distance of approximately 14 cm from the active electrode; (2) difference between the median and ulnar nerve distal sensory latencies is <0.4 ms; (3) the upper limit of distal latency of the median motor nerve is <4.3 ms at a distance of approximately 8 cm from the thenar muscles^20^,^21^,^22^. Furthermore, patients with symptoms mimicking CTS, such as polyneuropathy, brachial plexopathy, thoracic outlet syndrome, and history of wrist surgery or steroid injection for CTS were all excluded.

### Severity of electrophysiological CTS

Based on the electrophysiological classification report by Padua *et al*., we only recruited patients with mild to moderate CTS (Minimal: abnormal segmental or comparative tests only; Mild: only abnormal digit/wrist sensory nerve conduction velocity with normal distal motor latency; Moderate: abnormal digit/wrist sensory nerve conduction velocity and abnormal distal motor latency; Severe: absence of sensory response and abnormal distal motor latency; Extreme: absence of motor and sensory responses^22^.

### Shock wave therapy instrumentation

Patients were seated in a relaxed position with their forearm and finger placed on the table. The median nerve was identified at the pisiform level of the proximal carpal tunnel, with the palm facing upwards, by one physician using musculoskeletal ultrasonography (Neurotherm NT1000, Neurotherm Inc., USA)^11^,^23^,^24^. rESWT was delivered with the Physio Shock Wave Therapy system (Pagani Elettronica, Milano, Italy)^11^. The rESWT probe was located and oriented perpendicularly on the median nerve, and treatment comprised 2000 shots at a bar pressure of 4 and a pulse repetition frequency of 5 Hz for each patient^11^. The treated area was parallel to the median nerve from the pisiform level to 2 cm proximal to the inlet of the carpal tunnel with equal diffusion of 2000 shots. The procedure was painless, and there was no need for additional anesthesia or analgesia. In the control group, sham rESWT just made the same sound without energy emission.

### Outcome measurements

**Figure 1:**
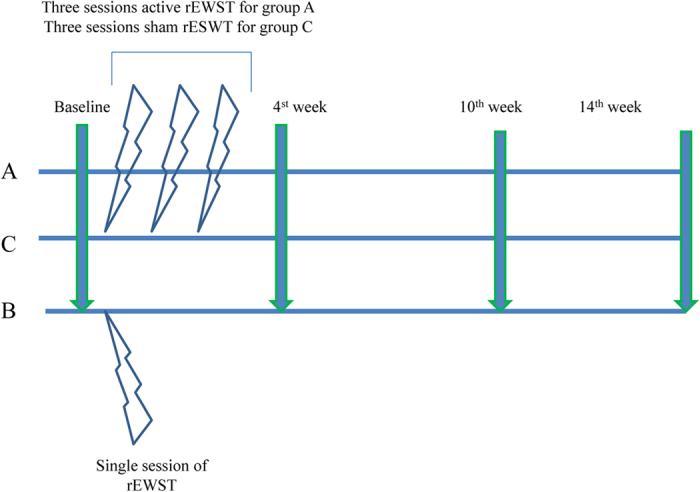
Timeline of treatment session with data collection in the three groups. Group A patients received one session of radial extracorporeal shock wave therapy (rESWT) per week for 3 consecutive weeks; Group B patients received a single session of rESWT; Group C patients received one session of sham rESWT per week for 3 consecutive weeks. The night splint was given in all patients since first-session of rESWT. Evaluations were performed before the first rESWT treatment, and at 4, 10, and 14 weeks after the first session of rESWT in each group.

The same physiatrist who was blinded to the randomization and treatment procedures carried out all the outcome measurements. Evaluations were performed before the first treatment of rESWT, and at the 4^th^, 10^th,^ and 14^th^ weeks after the first session of rESWT (Fig. 1).

### Primary outcome

#### Boston Carpal Tunnel Syndrome Questionnaire (BCTQ)

BCTQ is the most commonly used questionnaire for the measurement of the severity of symptoms and functional status with reproducibility, internal consistency, and validity in patients with CTS^25^. The symptom severity subscale consists of 11 questions with scores from 1 point (mildest) to 5 points (most severe), and the functional status subscale is made up of 8 questions with scores from 1 point (no difficulty in activity) to 5 points (unable to perform the activity at all).

### Secondary outcomes

#### Sensory nerve conduction velocity (SNCV)

Examination of the antidromic SNCV of the median nerve was performed on all participants based on the procedure protocol reported by the American Academy of Neurology with SierraWave, Cadwell (USA)^26^. All the evaluations were executed in the same room with the temperature kept at 25 °C by the same physiatrist. The surface temperature of the tested hand and wrist was maintained between 32 °C and 34 °C. The active and reference ring electrodes were placed over the 2^nd^ proximal and distal interphalangeal joints. The sensory nerve conduction study was performed by stimulating the median nerve at the wrist between the palmaris longus and flexor carpi radialis tendon, approximately 14 cm proximal to the active electrode. All the parameters were measured three times, and the mean value was used for the statistical analysis.

#### Cross-sectional area (CSA) of median nerve

Measurement of the CSA of the median nerve was performed at the proximal inlet of the carpal tunnel (parallel with the pisiform bone) by the same physiatrist. The wrist was placed on the table in a neutral position, with palm up and fingers extended. The CSA was measured using electronic calipers and calculated three times, and the mean value was used for statistical analysis. The CSA has been reported to have high sensitivity (89%) and specificity (83%) for the diagnosis of CTS^11^,^23^,^24^.

### Sample size

To reduce a type II error, a preliminary power analysis using G*power 3.1.9.2 computer program, based on a One-way analysis of variance (ANOVA) test with comparison of the 3 groups; power (1-β) = 0.9; α = 0.05; effect size = 0.4, indicated that a total sample of 84 wrists would be needed^27^.

### Data analysis

All the data were statistically analyzed by using the IBM SPSS statistics version 22 (IBM^®^ SPSS^®^ statistics 22). Demographic data were analyzed by the One-way ANOVA test for continuous data and the Chi-square test/Fisher’s exact test for categorical data. Differences between the 3 groups were investigated using the One-way ANOVA followed by the Bonferroni post hoc tests. Statistical significance was set at p < 0.05.

## Results

**Figure 2:**
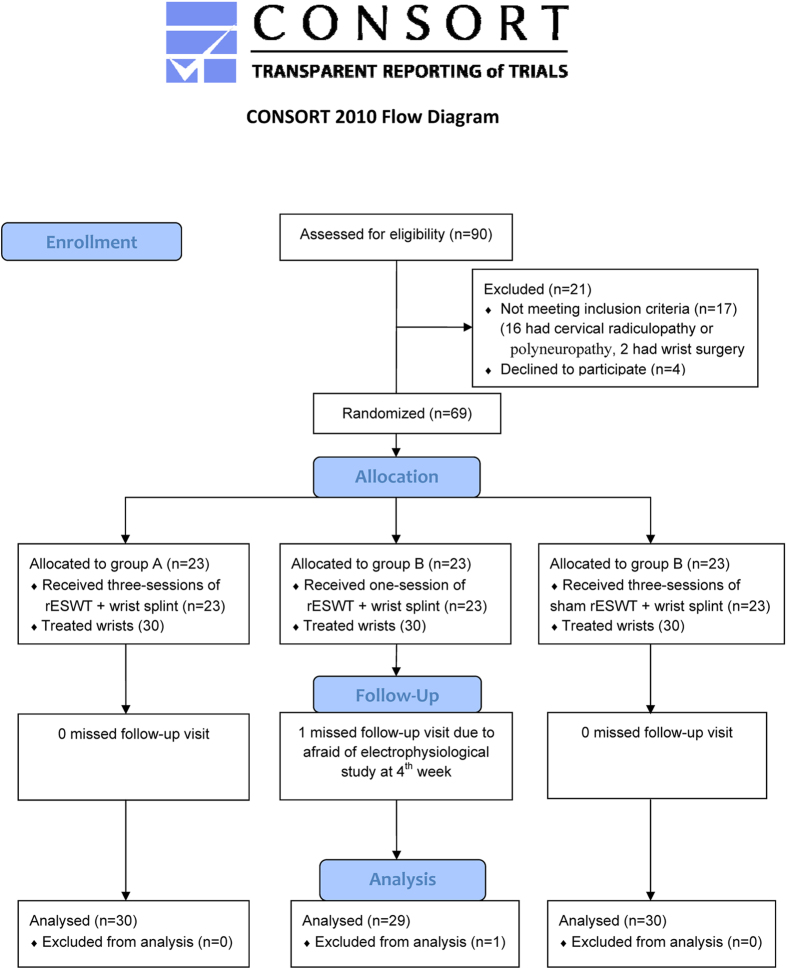
CONSORT flow diagram^16^.

A total of 68 patients completed the study with 30, 29, and 30 wrists in groups A, B, and C respectively (Fig. 2). There were no adverse effects or complications after rESWT application in any of the three groups during the study period.

Table 1 shows the baseline demographic and clinical characteristics between the three groups with no significant differences. Table 2 shows the differences in BCTQ, SNCV, and CSA scores before and after treatment in each group. The differences in both BCTQ subscales scores in group A were significantly larger than those of groups B and C at all observed time-points (p < 0.05). However, there was no significant difference in the BCTQ scores (severity or function) between group B and group C. Although there was a much greater improvement in SNCV, except in week 4, between groups A and C, groups B and C, and groups A and B, the differences did not reach significance. Similar findings were found in CSA, though the difference reached significance at week 14 between groups A and C.

**Figure 3:**
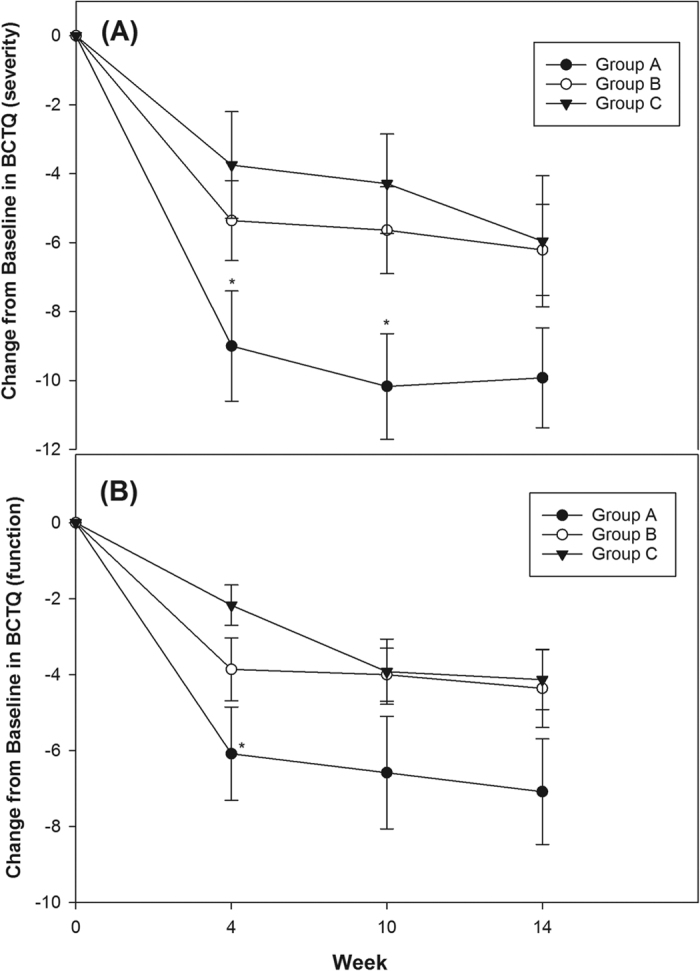
Mean change from baseline in Boston Carpal Tunnel Syndrome Questionnaire (BCTQ) in all groups in patients with mild CTS (mean ± standard error). (**A**) BCTQ of severity: Group A had significant improvement compared with group C until week 10. (**B**) BCTQ of function: Group A had significant improvement compared with group C until week 4. Although a tendency towards reduced BCTQ (severity and function) was found in group A vs B, and group B vs. C, the discrepancy did not reach significance. (*p < 0.05, One-way ANOVA followed by the Bonferroni post hoc tests was used).

**Figure 4:**
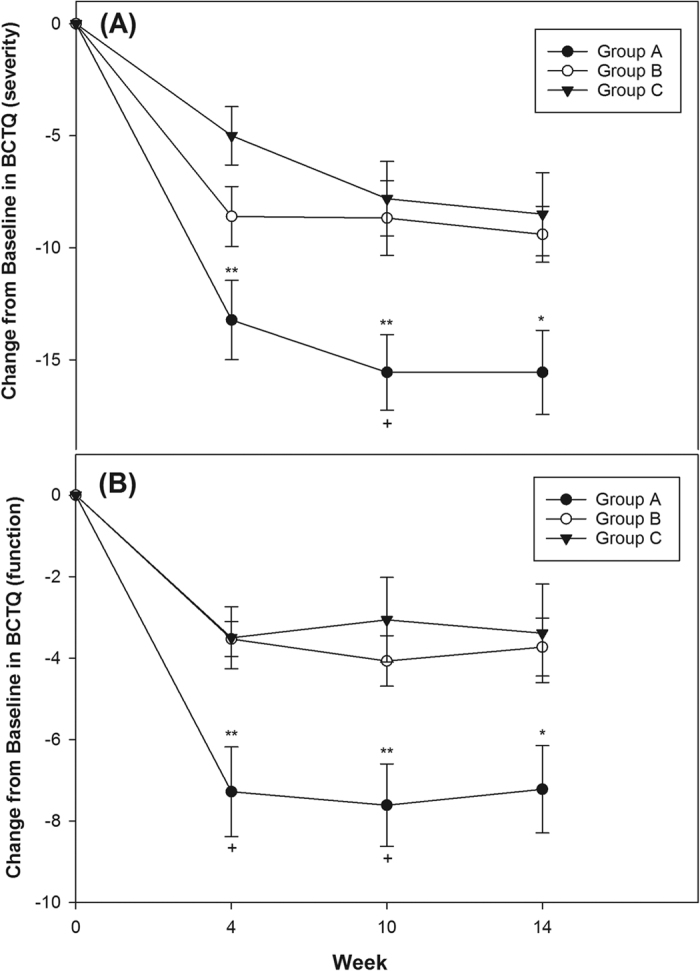
Mean of change from baseline in Boston Carpal Tunnel Syndrome Questionnaire (BCTQ) in all groups in patients with moderate CTS. (**A**) BCTQ of severity: Group A had significant improvement compared with group B and group C until week 10 and week 14 respectively. (**B**) BCTQ of function: Group A had significant improvement compared with group B and group C until week 10 and week 14 respectively. Although a tendency toward a reduced BCTQ (severity and function) was found in group B compared with group C, the discrepancy did not reach significance. (*p < 0.05, **p < 0.01 mean Group A vs. C; ^+^p < 0.05 mean Group A vs. B. One-way ANOVA followed by the Bonferroni post hoc tests was used).

We further separately analyzed the mild and moderate subgroups with 12, 14, and 12 wrists, and 18, 15, and 18 wrists graded as mild or moderate CTS within the groups respectively (Table 3, Figs 3 and 4). Although there was a much greater improvement in SNCV and CSA between groups A and C, groups B and C, and groups A and B at most observed time-points with regard to mild and moderate CTS, the differences did not reach significance, except between groups A and C in CSA at week 14 (Table 3). Regarding the BCTQ, the differences in both BCTQ severity and function scores in group A were significantly bigger than those in group C until week 10, and week 4 in the mild CTS subgroup (Fig. 3). Similar findings in both BCTQ scores were observed in the moderate CTS subgroup, and the significance extended to week 14. Furthermore, there was significant enhancement between groups A and B with respect to BCTQ (severity and function) until week 10 (Fig. 4). Although a tendency towards lower BCTQ scores was found in group B compared with group C in both mild and moderate CTS subgroups, this discrepancy did not reach significance (Figs 3 and 4).

## Discussion

To the best of our knowledge, the present study is the first prospective, randomized, single-blind, placebo-controlled study to investigate the dose-dependent efficiency of rESWT for treating patients with mild to moderate CTS. Compared to the control group, three-sessions of rESWT in the intervention group demonstrated significant pain and disability reduction at least 14 weeks after the first session of rESWT, and the effect was more noticeable and longer-lasting in patients with moderate CTS than mild CTS. In contrast, the effect of single-session rESWT is insignificant for both mild and moderate CTS compared with the control group.

Recently, the clinical effect of ESWT on peripheral nerves has received more attention. A few studies have tried to apply ESWT as an alternative management for treating peripheral neuropathy, for example interdigital neuroma^28^, stump neuroma^29^, distal symmetric polyneuropathy^30^, and CTS^13^,^14^,^15^, with inspiring results. In 2013, Seok *et al*.^13^ first reported that one-session fESWT could be as useful as corticosteroid injection for relieving symptoms of CTS (n = 15 vs. n = 16 respectively), and that the effect could persist for at least 3 months. A subsequent study by Notarnicola *et al*.^15^ showed at least a six month effect of combined 3-session fESWT with splint or nerve/tendon-gliding exercises compared with a nutraceutical composed of Echinacea angustifolia, alpha lipoic acid, conjugated linoleic acid and quercetin in patients with CTS (n = 34 vs. n = 26 respectively). Paoloni *et al*.^14^ also reported that patients with mild to moderate CTS might benefit in terms of pain and disability from 3 session of fESWT alone compared with ultrasound and cryo-ultrasound therapy, and that the effect could persist for 3 months (n = 12 vs. n = 13 vs. n = 17, respectively). In 2015, we first studied rESWT with a prospective, randomized, double-blind, placebo-controlled study, and revealed the benefit of rESWT in treating CTS after 3 months follow-up (n = 20 vs. n = 20, respectively)^11^. However, current studies enrolled small patient numbers and most were lacking placebo control or blind design. Moreover, these studies did not further notify us of the diverse effect of ESWT in different grades of CTS. Additionally, several questions, including the most effective intensity and number of ESWT sessions, remained unanswered.

Our study, which recruited more patient numbers, has proven the therapeutic effect of rESWT in mild to moderate CTS once again. Specifically, we have, for the first time, demonstrated that the effect of rESWT is greater in moderate CTS compared to mild CTS. Mild CTS partially resolves spontaneously and has more potential improvement with adequate conservative treatments, such as night splint, compared with moderate CTS, and this might explain the different effects of rESWT in different grades of CTS. The splint is the most popular method to treat mild to moderate CTS with modest evidence of short-term effect^6^. The night splint in our study had good effect for mild CTS, therefore the results of bonus ESWT would not be worth mentioning.

Standard guidelines for the use of ESWT have not been established. Nevertheless, numerous studies used 2 or more sessions of ESWT for chronic musculoskeletal disorders. Thus, clinical experience indicates repeated sessions of ESWT could be superior to a single application. Although we have proven the cumulative effect of rESWT in stroke patients with chronic spasticity^31^, the mechanism of peripheral neuropathy was far different from musculoskeletal disorders or spasticity. Takahashi *et al*. revealed low-energy ESWT could have a cumulative effect on free nerve endings of rats with a longer-lasting antinociceptive result after multiple attempts^32^. Whether the cumulative clinical effect of ESWT would occur in peripheral neuropathy is unknown. The results in our study firstly verify the cumulative effect of rESWT for mild to moderate CTS. In addition, Fu *et al*.^33^ have shown that a single-session rESWT only has a 5-day efficacy over mechanical hyperalgesia and thermal hyperalgesia in the rat, and that repeated sessions of rESWT could maintain the analgesic effect for at least 4 weeks. This might explain why we did not observe a noteworthy benefit after single-session rESWT compared with sham rESWT, although much greater improvements in BCTQ, most SNCV and CSA were observed. Most previous studies applied 3 sessions of ESWT in treating CTS except the study by Seok *et al*.^13^, which used single-session fESWT. Differences in the mechanism used to generate the shock wave, therapeutic energy, number of applications, duration of CTS, and patients’ age may have contributed to the variation in effect. Further study would have been encouraged to compare rESWT with fESWT.

The degree of improved SNCV (group C > B > A) between three groups caught our attention. Additional one- and three-session rESWT would contrarily reduce SNCV at week 4 after the first session of rESWT, and reversed to increase SNCV after week 10 compared with Group C (Tables 2 and 3). Also, repetitive rESWT resulted in more noticeable reductions in SNCV. Ohtori *et al*.^34^ observed nearly complete degeneration of intracutaneous nerve fibers after applying low-energy ESWT to rat skin on days 2, 4, and 7, and that re-innervation occurs 2 weeks after treatment without significant differences. Takahashi *et al*.^32^ confirmed similar findings and revealed that multiple sessions of low-energy ESWT would provide a longer-lasting effect. Hausner *et al*.^35^ reported that low-energy ESWT could induce a significant recovery of nerve regeneration and amplitude in rats treated with nerve autograft of the sciatic nerve, and that the effect is most remarkable at 8 weeks after the nerve injury. Wu *et al*.^36^,^37^ showed that although high-intensity ESWT (0.49 mJ/mm^2^) induced temporary 60–80% reductions in motor nerve conduction velocity (MNCV) of the sciatic nerve in rats, no significant change in functional activity was observed and the reduced MNCV recovered within 14 days after treatment. In addition, they found the decline in MNCV persisted longer with higher intensity of ESWT. Our findings might be compatible with the above experimental studies. However, current clinical studies are insufficient to identify the hypothesis, and further studies are needed.

The definite mechanism behind the effects of ESWT on peripheral neuropathy is still not clear. The ESWT would stimulate the production of endothelial nitric oxides (NO), angiogenesis, and neurogenesis through vascular endothelial growth factor (VEGF)^38^,^39^,^40^,^41^,^42^. Moreover, ESWT might reduce calcitonin gene–related peptide (CGRP) with accompanying anti-inflammatory effects in the median nerve and its surrounding soft tissue^11^,^13^,^43^. Moreover, the temporarily reduced SNCV soon after rESWT in both groups A and B compared with group C may prove the hypothesis that rapid degeneration of intracutaneous nerve fibers might result in pain relief after ESWT^34^.

There are some limitations to this study. First, the mechanism of rESWT for CTS was not evaluated in this study. Second, it would be better to follow up patients for a longer time. Significant improvement of BCTQ and CSA scores was observed in week 14 in group A compared with that in group C. Hence, we believe the effect could continue for more than 14 weeks if the follow-up period was extended. Third, another double session of sham EWST in group B would add validity to our study. Finally, SNCV of the median nerve alone cannot engender the detailed information from electrophysiological studies, and it would be better to record MNCV too. Further study is expectant.

In conclusion, our findings suggest that rESWT is a valuable and novel strategy for patients with mild to moderate CTS, and that the effect was more noticeable and longer-lasting in patients with moderate CTS. In addition, multiple-session rESWT would result in a cumulative clinical effect. In contrast, the effect of single-session rESWT is insignificant for both mild and moderate CTS. Although additional, larger, longer-term studies are needed to confirm our results, this simple and repeatable procedure highlights the potential of ESWT as a new approach in treating CTS.

## Additional Information

**How to cite this article**: Ke, M.-J. *et al*. The dose-dependent efficiency of radial shock wave therapy for patients with carpal tunnel syndrome: a prospective, randomized, single-blind, placebo-controlled trial. *Sci. Rep.* **6**, 38344; doi: 10.1038/srep38344 (2016).

**Publisher's note:** Springer Nature remains neutral with regard to jurisdictional claims in published maps and institutional affiliations.

## Figures and Tables

**Table 1:** Baseline demographic and clinical characteristics of all study participants (mild and moderate).

	Group A (n = 30)	Group B (n = 29)	Group C (n = 30)	^a^*p* value
Gender, n (%)				0.915
Male	6 (20%)	6 (20.69%)	5 (16.67%)	
Female	24 (80%)	23 (79.31%)	25 (83.33%)	
Age (year) ± SE (Range)	56.33 ± 1.48 (37–71)	55.45 ± 1.38 (40–68)	58.13 ± 1.13 (45–66)	0.356
BH (cm) ± SE (Range)	158.20 ± 1.41 (143–172)	159.03 ± 1.31 (147–172)	155.90 ± 1.09 (145–168)	0.204
BW (kg) ± SE (Range)	64.56 ± 2.47 (43–100)	62.62 ± 2.39 (47–95)	63.53 ± 1.81 (45–88)	0.830
DM, n (%)				0.622
No	29 (96.67%)	26 (89.66%)	27 (90%)	
Yes	1 (3.33%)	3 (10.34%)	3 (10%)	
HTN, n (%)				0.621
No	24 (80%)	20 (68.97%)	22 (73.33%)	
Yes	6 (20%)	9 (31.03%)	8 (26.67%)	
Lesion site, n (%)				0.865
Right	14 (46.67%)	15 (51.72%)	16 (53.33%)	
Left	16 (53.33%)	14 (48.28%)	14 (46.67%)	
Duration (month) ± SE (Range)	34.27 ± 5.85 (4–120)	35.34 ± 7.45 (4–120)	34.37 ± 5.42 (6–120)	0.991

Group A: 3 sessions of active rESWT; Group B: 1 session of active rESWT; Group C: 3 sessions of sham rESWT.

SE = Standard error, BH = Body height, BW = Body weight, DM = Diabetes mellitus, HTN = Hypertension.

^a^One-way ANOVA test or Chi-square test/Fisher’s exact test.

**Table 2:** Mean and SE of change from baseline of all study participants (mild and moderate).

	Group A (n = 30) Difference (Mean ± SE)	Group B (n = 29) Difference (Mean ± SE)	Group C (n = 30) Difference (Mean ± SE)	(Across 3 groups)^a^*P* value	(Between 2 groups)^b^*P* value
BCTQs (Baseline)	28.70 ± 1.33	24.69 ± 1.41	26.87 ± 1.54	0.147	
WK4-Baseline	−11.53 ± 1.28	−7.03 ± 0.93	−4.50 ± 0.98	<0.001	A vs. B* A vs. C*** B vs. C
WK10-Baseline	−13.40 ± 1.26	−7.21 ± 1.08	−6.40 ± 1.18	<0.001	A vs. B** A vs. C*** B vs. C
WK14-Baseline	−13.30 ± 1.35	−7.86 ± 0.94	−7.48 ± 1.35	0.002	A vs. B** A vs. C** B vs. C
BCTQf (Baseline)	17.30 ± 0.96	15.17 ± 0.87	16.30 ± 0.85	0.250	
WK4-Baseline	−6.80 ± 0.82	−3.69 ± 0.45	−2.97 ± 0.51	<0.001	A vs. B** A vs. C*** B vs. C
WK10-Baseline	−7.20 ± 0.84	−4.03 ± 0.46	−3.40 ± 0.70	<0.001	A vs. B** A vs. C** B vs. C
WK14-Baseline	−7.17 ± 0.83	−4.03 ± 0.61	−3.68 ± 0.79	0.002	A vs. B* A vs. C** B vs. C
SNCV (m/s) (Baseline)	31.81 ± 1.08	34.17 ± 1.23	32.77 ± 1.33	0.394	
WK4-Baseline	1.04 ± 0.26	1.44 ± 0.29	1.82 ± 0.33	0.172	—
WK10-Baseline	2.16 ± 0.42	1.83 ± 0.21	1.92 ± 0.30	0.760	—
WK14-Baseline	3.09 ± 0.51	2.50 ± 0.30	2.03 ± 0.40	0.189	—
CSA (mm^2^) (Baseline)	13.19 ± 0.40	12.26 ± 0.41	12.67 ± 0.37	0.261	
WK4-Baseline	−1.61 ± 0.16	−1.40 ± 0.22	−1.04 ± 0.17	0.088	—
WK10-Baseline	−1.97 ± 0.21	−1.52 ± 0.20	−1.29 ± 0.19	0.060	—
WK14-Baseline	−2.34 ± 0.25	−1.82 ± 0.24	−1.36 ± 0.21	0.015	A vs. B A vs. C* B vs. C

Group A: 3 sessions of active rESWT; Group B: 1 session of active rESWT; Group C: 3 sessions of sham rESWT.

SE = Standard error, WK = Week, rESWT = Radial Extracorporeal shock wave therapy, BCTQ = Boston Carpal Tunnel Syndrome Questionnaire (s = severity and f = function); SNCV = Sensory nerve conduction velocity; CSA = Cross-sectional area.

*p < 0.05, **p < 0.01, ***p < 0.001.

^a^One-way ANOVA test.

^b^Bonferroni post hoc tests.

**Table 3:** Mean and SE of change from baseline of mild and moderate carpal tunnel syndrome.

Mild	Group A (n = 12) Difference (Mean ± SE)	Group B (n = 14) Difference (Mean ± SE)	Group C (n = 12) Difference (Mean ± SE)	(Across 3 groups)^a^ *P* value	(Between 2 groups)^b^ *P* value
SNCV (m/s) (Baseline)	37.56 ± 0.89	38.93 ± 0.49	39.66 ± 0.74	0.127	
WK4-Baseline	1.05 ± 0.30	1.33 ± 0.51	1.88 ± 0.54	0.474	
WK10-Baseline	1.84 ± 0.58	1.72 ± 0.38	1.80 ± 0.38	0.981	
WK14-Baseline	2.91 ± 0.75	2.16 ± 0.48	1.85 ± 0.45	0.423	
CSA (mm^2^) (Baseline)	11.94 ± 0.68	10.84 ± 0.39	11.63 ± 0.67	0.380	
WK4-Baseline	−1.74 ± 0.25	−1.23 ± 0.25	−1.17 ± 0.18	0.185	
WK10-Baseline	−2.02 ± 0.35	−1.42 ± 0.26	−1.53 ± 0.33	0.359	
WK14-Baseline	−2.32 ± 0.34	−1.55 ± 0.30	−1.76 ± 0.37	0.259	
**Moderate**	**Group A (n = 18) Difference (Mean ± SE)**	**Group B (n = 15) Difference (Mean ± SE)**	**Group C (n = 18) Difference (Mean ± SE)**	**(Across 3 groups)** ^**a**^***P*** **value**	**(Between 2 groups)** ^**b**^***P*** **value**
SNCV (m/s) (Baseline)	27.99 ± 0.89	29.73 ± 1.64	28.18 ± 1.32	0.596	
WK4-Baseline	1.03 ± 0.38	1.53 ± 0.31	1.77 ± 0.42	0.360	—
WK10-Baseline	2.38 ± 0.59	1.93 ± 0.23	2.00 ± 0.45	0.771	—
WK14-Baseline	3.21 ± 0.69	2.82 ± 0.36	2.14 ± 0.60	0.420	—
CSA (mm^2^) (Baseline)	14.02 ± 0.41	13.59 ± 0.52	13.37 ± 0.36	0.530	
WK4-Baseline	−1.53 ± 0.21	−1.56 ± 0.37	−0.96 ± 0.26	0.216	—
WK10-Baseline	−1.94 ± 0.27	−1.61 ± 0.32	−1.13 ± 0.24	0.111	—
WK14-Baseline	−2.35 ± 0.36	−2.08 ± 0.37	−1.09 ± 0.25	0.019	A vs. B A vs. C* B vs. C

Group A: 3 sessions of active rESWT; Group B: 1 session of active rESWT; Group C: 3 sessions of sham rESWT.

SE = Standard error, WK = Week, rESWT = Radial Extracorporeal shock wave therapy, SNCV = Sensory nerve conduction velocity, CSA = Cross-sectional area.

*p < 0.05, **p < 0.01, ***p < 0.001.

^a^One-way ANOVA test.

^b^Bonferroni post hoc tests.

## References

[b1] Werner R. A. & Andary M. Carpal tunnel syndrome: pathophysiology and clinical neurophysiology. Clin Neurophysiol. 113, 1373–1381 (2002). 12169318 10.1016/s1388-2457(02)00169-4

[b2] Atroshi I. . Prevalence for clinically proved carpal tunnel syndrome is 4 percent. Lakartidningen. 97, 1668–1670 (2000). 10815392

[b3] van Dijk M. A., Reitsma J. B., Fischer J. C. & Sanders G. T. Indications for requesting laboratory tests for concurrent diseases in patients with carpal tunnel syndrome: a systematic review. Clin Chem. 49, 1437–1444 (2003). 12928223 10.1373/49.9.1437

[b4] Gelberman R. H., Hergenroeder P. T., Hargens A. R., Lundborg G. N. & Akeson W. H. The carpal tunnel syndrome. A study of carpal canal pressures. J Bone Joint Surg Am. 63, 380–383 (1981). 7204435

[b5] de Krom M. C., van Croonenborg J. J., Blaauw G., Scholten R. J. & Spaans F. Guideline ‘Diagnosis and treatment of carpal tunnel syndrome’. Ned Tijdschr Geneeskd. 152, 76–81 (2008). 18265795

[b6] Huisstede B. M. . Carpal tunnel syndrome. Part I: effectiveness of nonsurgical treatments–a systematic review. Arch Phys Med Rehabil. 91, 981–1004 (2010). 20599038 10.1016/j.apmr.2010.03.022

[b7] Ahcan U., Arnez Z. M., Bajrović F. & Zorman P. Surgical technique to reduce scar discomfort after carpal tunnel surgery. J Hand Surg Am. 27, 821–827 (2002). 12239671 10.1053/jhsu.2002.35083

[b8] Akhtar S. . Study to assess differences in outcome following open carpal tunnel decompressions performed by surgeons of differing grade. Ann R Coll Surg Engl. 89, 785–788 (2007). 17999820 10.1308/003588407X232035 PMC2173181

[b9] Katz J. N. . Maine Carpal Tunnel Study: outcomes of operative and nonoperative therapy for carpal tunnel syndrome in a community-based cohort. J Hand Surg Am. 23, 697–710 (1998). 9708386 10.1016/S0363-5023(98)80058-0

[b10] Rompe J. D., Decking J., Schoellner C. & Nafe B. Shock wave application for chronic plantar fasciitis in running athletes. A prospective, randomized, placebo-controlled trial. Am J Sports Med. 31, 268–275 (2003). 12642264 10.1177/03635465030310021901

[b11] Wu Y. T. . Effect of radial shock wave therapy for carpal tunnel syndrome: A prospective randomized, double-blind, placebo-controlled trial. J Orthop Res. 26 [Epub ahead of print] (2015). 10.1002/jor.23113 26610183

[b12] Gerdesmeyer L. . Extracorporeal shock wave therapy for the treatment of chronic calcifying tendonitis of the rotator cuff: a randomized controlled trial. JAMA. 290, 2573–2580 (2003). 14625334 10.1001/jama.290.19.2573

[b13] Seok H. & Kim S. H. The effectiveness of extracorporeal shock wave therapy vs. local steroid injection for management of carpal tunnel syndrome: a randomized controlled trial. Am J Phys Med Rehabil. 92, 327–334 (2013). 23044704 10.1097/PHM.0b013e31826edc7b

[b14] Paoloni M. . Extracorporeal shock wave therapy and ultrasound therapy improve pain and function in patients with carpal tunnel syndrome. A randomized controlled trial. Eur J Phys Rehabil Med. 51, 521–528 (2015). 25697763

[b15] Notarnicola A. . Comparison of shock wave therapy and nutraceutical composed of Echinacea angustifolia, alpha lipoic acid, conjugated linoleic acid and quercetin (perinerv) in patients with carpal tunnel syndrome. Int J Immunopathol Pharmacol. 28, 256–262 (2015). 25953494 10.1177/0394632015584501

[b16] Moher D. . CONSORT 2010 explanation and elaboration: updated guidelines for reporting parallel group randomised trials. BMJ. 340, c869 (2010). 20332511 10.1136/bmj.c869 PMC2844943

[b17] O’Connor D., Marshall S. & Massy-Westropp N. Non-surgical treatment (other than steroid injection) for carpal tunnel syndrome. Cochrane Database Syst Rev. CD003219 (2003). 10.1002/14651858.CD003219 PMC6486195 12535461

[b18] Jablecki C. K. . Practice parameter: Electrodiagnostic studies in carpal tunnel syndrome. Report of the American Association of Electrodiagnostic Medicine, American Academy of Neurology, and the American Academy of Physical Medicine and Rehabilitation. Neurology. 58, 1589–1592 (2002). 12058083 10.1212/wnl.58.11.1589

[b19] You H., Simmons Z., Freivalds A., Kothari M. J. & Naidu S. H. Relationships between clinical symptom severity scales and nerve conduction measures in carpal tunnel syndrome. Muscle Nerve. 22, 497–501 (1999). 10204785 10.1002/(sici)1097-4598(199904)22:4<497::aid-mus11>3.0.co;2-t

[b20] Rossi S. . Sensory neural conduction of median nerve from digits and palm stimulation in carpal tunnel syndrome. Electroencephalogr Clin Neurophysiol. 93, 330–334 (1994). 7525240 10.1016/0168-5597(94)90120-1

[b21] Padua L., Lo Monaco M., Valente E. M. & Tonali P. A. A useful electrophysiologic parameter for diagnosis of carpal tunnel syndrome. Muscle Nerve. 19, 48–53 (1996). 8538669 10.1002/(SICI)1097-4598(199601)19:1<48::AID-MUS6>3.0.CO;2-8

[b22] Padua L. . Neurophysiological classification and sensitivity in 500 carpal tunnel syndrome hands. Acta Neurol Scand. 96, 211–217 (1997). 9325471 10.1111/j.1600-0404.1997.tb00271.x

[b23] Wong S. M., Griffith J. F., Hui A. C., Tang A. & Wong K. S. Discriminatory sonographic criteria for the diagnosis of carpal tunnel syndrome. Arthritis Rheum. 46, 1914–1921 (2002). 12124876 10.1002/art.10385

[b24] Chen L. C. . Ultrasound-guided pulsed radiofrequency for carpal tunnel syndrome: a single-blinded randomized controlled study. PLoS ONE 10, e0129918 (2015). 26067628 10.1371/journal.pone.0129918 PMC4466776

[b25] Levine D. W. . A self-administered questionnaire for the assessment of severity of symptoms and functional status in carpal tunnel syndrome. J Bone Joint Surg Am. 75, 1585–1592 (1993). 8245050 10.2106/00004623-199311000-00002

[b26] Jablecki C. K., Andary M. T., So Y. T., Wilkins D. E. & Williams F. H. Literature review of the usefulness of nerve conduction studies and electromyography for the evaluation of patients with carpal tunnel syndrome. AAEM Quality Assurance Committee. Muscle Nerve. 16, 1392–1414 (1993). 8232399 10.1002/mus.880161220

[b27] Faul F., Erdfelder E., Lang A. G. & Buchner A. G*Power 3: a flexible statistical power analysis program for the social, behavioral, and biomedical sciences. Behav Res Methods 39, 175–191 (2007). 17695343 10.3758/bf03193146

[b28] Fridman R., Cain J. D. & Weil L. J. Extracorporeal shockwave therapy for interdigital neuroma: a randomized, placebo-controlled, double-blind trial. J Am Podiatr Med Assoc. 99, 191–193 (2009). 19448168 10.7547/0980191

[b29] Jung Y. J. . Outcomes of ultrasound-guided extracorporeal shock wave therapy for painful stump neuroma. Ann Rehabil Med. 38, 523–533 (2014). 25229031 10.5535/arm.2014.38.4.523 PMC4163592

[b30] Lohse-Busch H., Marlinghaus E., Reime U. & Möwis U. Focused low-energy extracorporeal shock waves with distally symmetric polyneuropathy (DSPNP): a pilot study. Neuro-Rehabilitation 35, 227–233 (2014). 24990024 10.3233/NRE-141116

[b31] Li T. Y. . Effect of radial shock wave therapy on spasticity of the upper limb in patients with chronic stroke: A prospective, randomized, single blind, controlled trial. Medicine 95, e3544 (2016). 27149465 10.1097/MD.0000000000003544 PMC4863782

[b32] Takahashi N., Ohtori S., Saisu T., Moriya H. & Wada Y. Second application of low-energy shock waves has a cumulative effect on free nerve endings. Clin Orthop Relat Res. 443, 315–319 (2006). 16462457 10.1097/01.blo.0000188064.56091.a7

[b33] Fu M. . Radial shock wave therapy in the treatment of chronic constriction injury model in rats: a preliminary study. Chin Med J (Engl). 127, 830–834 (2014). 24571871

[b34] Ohtori S. . Shock wave application to rat skin induces degeneration and reinnervation of sensory nerve fibres. Neurosci Lett. 315, 57–60 (2001). 11711214 10.1016/s0304-3940(01)02320-5

[b35] Hausner T. . Improved rate of peripheral nerve regeneration induced by extracorporeal shock wave treatment in the rat. Exp Neurol. 236, 363–370 (2012). 22575596 10.1016/j.expneurol.2012.04.019

[b36] Wu Y. H. . Electrophysiological and functional effects of shock waves on the sciatic nerve of rats. Ultrasound Med Biol. 34, 1688–1696 (2008). 18468775 10.1016/j.ultrasmedbio.2008.03.005

[b37] Wu Y. H., Lun J. J., Chen W. S. & Chong F. C. The electrophysiological and functional effect of shock wave on peripheral nerves. Conf Proc IEEE Eng Med Biol Soc. 2007, 2369–2372 (2007). 18002469 10.1109/IEMBS.2007.4352803

[b38] Mariotto S. . Extracorporeal shock waves: from lithotripsy to anti-inflammatory action by NO production. Nitric Oxide 12, 89–96 (2005). 15740982 10.1016/j.niox.2004.12.005

[b39] Ito K., Fukumoto Y. & Shimokawa H. Extracorporeal shock wave therapy as a new and non-invasive angiogenic strategy. Tohoku J Exp Med. 219, 1–9 (2009). 19713678 10.1620/tjem.219.1

[b40] Stojadinovic A. . Angiogenic response to extracorporeal shock wave treatment in murine skin isografts. Angiogenesis 11, 369–380 (2008). 18998221 10.1007/s10456-008-9120-6

[b41] Jin K., Mao X. O. & Greenberg D. A. Vascular endothelial growth factor stimulates neurite outgrowth from cerebral cortical neurons via Rho kinase signaling. J Neurobiol. 66, 236–242 (2006). 16329123 10.1002/neu.20215

[b42] Sun Y. . Vascular endothelial growth factor-B (VEGFB) stimulates neurogenesis: evidence from knockout mice and growth factor administration. Dev Biol. 289, 329–335 (2006). 16337622 10.1016/j.ydbio.2005.10.016

[b43] Takahashi N., Wada Y., Ohtori S., Saisu T. & Moriya H. Application of shock waves to rat skin decreases calcitonin gene-related peptide immunoreactivity in dorsal root ganglion neurons. Auton Neurosci. 107, 81–84 (2003). 12963418 10.1016/S1566-0702(03)00134-6

